# Supplementary material for: Differences in the optical properties of photochromic lenses between cold and warm temperatures
Source: PLoS One. 2020 May 29;15(5):e0234066. doi: 10.1371/journal.pone.0234066 (PMC7259785; doi:10.1371/journal.pone.0234066)
Supplement: Attachment — Submitted filename: Response to Reviewers.docx [file pone.0234066.s003.docx]

**Editor’ Comments:**

The manuscript has been evaluated by three reviewers, and their comments are available below.

The reviewers have raised a number of concerns that need attention. They request additional information on methodological aspects of the study and have made several points be clarified in the text.

Could you please revise the manuscript to carefully address the concerns raised?

Response to Editor’ comments: We thank you and the reviewers for reviewing our manuscript and the valuable comments provided. We have revised our manuscript accordingly and provided point-by-point responses to the reviewers’ comments.

**Journal Requirements:**

(Requirements and Reviewer’s comments are shown in *Italic*)

*When submitting your revision, we need you to address these additional requirements.*

*1. Please ensure that your manuscript meets PLOS ONE's style requirements, including those for file naming. The PLOS ONE style templates can be found at*

*http://www.journals.plos.org/plosone/s/file?id=wjVg/PLOSOne_formatting_sample_main_body.pdf and http://www.journals.plos.org/plosone/s/file?id=ba62/PLOSOne_formatting_sample_title_authors_affiliations.pdf*

Response to Journal requirements (RJ1): We have verified and rechecked the manuscript to ensure that it meets the ‘Manuscript body formatting guidelines’ and ‘Title, author, affiliations formatting guidelines’.

**Reviewers’ Comments to the author:**

*1. Is the manuscript technically sound, and do the data support the conclusions?*

*The manuscript must describe a technically sound piece of scientific research with data that supports the conclusions. Experiments must have been conducted rigorously, with appropriate controls, replication, and sample sizes. The conclusions must be drawn appropriately based on the data presented.

Reviewer #1: Yes
Reviewer #2: Partly
Reviewer #3: Yes*

Authors’ Response: We have revised the manuscript and replied to the reviewers’ comments (R1R#2, R2R#2, R14R#2, R6R#2).

*2. Has the statistical analysis been performed appropriately and rigorously?

Reviewer #1: Yes
Reviewer #2: I Don't Know
Reviewer #3: Yes*

Authors’ Response: We have commented on R7R#2 related to the statistical data among the comments of reviewer #2.

*3. Have the authors made all data underlying the findings in their manuscript fully available?

The* [*PLOS Data policy*](http://www.plosone.org/static/policies.action#sharing) *requires authors to make all data underlying the findings described in their manuscript fully available without restriction, with rare exception (please refer to the Data Availability Statement in the manuscript PDF file). The data should be provided as part of the manuscript or its supporting information, or deposited to a public repository. For example, in addition to summary statistics, the data points behind means, medians and variance measures should be available. If there are restrictions on publicly sharing data—e.g. participant privacy or use of data from a third party—those must be specified.

Reviewer #1: Yes
Reviewer #2: Yes
Reviewer #3: Yes*

Authors’ Response: Thank you for the positive evaluation.

*4. Is the manuscript presented in an intelligible fashion and written in standard English?

PLOS ONE does not copyedit accepted manuscripts, so the language in submitted articles must be clear, correct, and unambiguous. Any typographical or grammatical errors should be corrected at revision, so please note any specific errors here.

Reviewer #1: Yes
Reviewer #2: No
Reviewer #3: Yes*

Authors’ Response: We have corrected any errors pointed out by the reviewers. Further, we have had our manuscript for resubmission edited by a native English speaker.

*5. Review Comments to the Author*

*Please use the space provided to explain your answers to the questions above. You may also include additional comments for the author, including concerns about dual publication, research ethics, or publication ethics. (Please upload your review as an attachment if it exceeds 20,000 characters)*

*Reviewer #1: It is well known that photochromic lenses darken more at medium and low temperatures (temperatures above zero). The conclusions of the research carried out by the authors confirm this phenomenon (see table 2. Optical properties of photochromic lenses./ Mean values of luminous transmittance for all tested samples (LT0)at warm temperature is 41% and in cold temperature is 31,0 %). The authors tested lenses conditioned at positive temperatures (6 and 12 oC). The choice of these temperatures is dictated by the climatic conditions in which protective glasses with mounted photochromic lenses are used. It would be interesting to carry out these tests for lenses conditioned at temperatures well below freezing. The practical aspect of the research is very important. I fully agree with the authors that information on changes in the selected optical properties of photochromic lenses (e.g. optical density, colored state and fading rate) should also be provided to users of glasses.*

Response 1 to Reviewer #1 (R1R#1): We appreciate the reviewer’s positive comments on our manuscript. We also thank the reviewer’s additional comments about an effect of a cooling below 0 °C on the lenses. Our experiment was not performed below 0 °C; however, as in the study of Look and Johnson [23], temperature-dependent transmittance had a linear relationship for the photochromic glass at the temperature range of -12.2–65.6 °C. Although our study was not conducted under similar conditions, if the temperature-dependent transmittance has a linear relationship, then the mean luminous transmittance at -6 °C would be anticipated to be approximately 24.8% for the photochromic lenses (Figure R1).

Please note that our response to the comment was not included in the manuscript because this is not the result of our experiments.

Figure R1. Predicted transmittance at temperature below freezing.

Reference:

23. Look DC, Johnson WL. Transmittance of photochromic glass at environmental extremes. Appl Opt. 1979; 18(5): 595–597.

*Reviewer #1: Suggestion to introduce small additions to the text:
The Authors write: “Other correlations were analyzed to examine the applicability of transmittance instead of luminous transmittance (LT) weighted by the photopic spectral sensitivity of the human eye at each wavelength”. To determine the value of luminous transmittance, the illuminant distribution is also taken into account. I would suggest writing the right formula in the Optical properties section or referring to the distribution of the illuminant in the text.*

R2R#1: The sentence ‘Luminous transmittance was calculated by the ratio of the luminous flux transmitted by the lens to the incident luminous flux [22].’ was added on page 8, line 167 in the Materials and methods section according to reviewer’s suggestion.

========================

*Reviewer #2: This paper reports an analysis of the photochromic behavior of commercial photochromic lenses with a focus on the effect of temperature on the performance of these systems. The authors use a big collection of gray and brown lenses from different manufacturers.*

R1R#2: We aimed at evaluating the photochromic characteristics according to temperature for commonly available photochromic lenses. We found that all photochromic lenses were significantly different with respect to their optical properties at the cold and warm temperature.

*Reviewer #2: The analysis of the results is quite detailed but the discussion is rather disorganized and difficult to follow. In particular, it would be very useful to add comparative graphics to follow the discussion which, for 16 samples, has just too many numbers. Also, the results and discussion parts should be divided into subsections and each section should include an objective conclusion.*

R2R#2: We reorganized the discussion section to improve readability. First, we checked the relation between the subsections of the results and the discussion. Second, we matched the subsections with the paragraphs. Third, we divided the paragraphs into subsections of the discussion, as shown in Figure R2. Finally, we presented and discussed the Tables and Figures related to the results in the subsections of the discussion.

Figure R2. Reorganized structure of discussion.

*Reviewer #2: The quality of the few available graphics is very low and they are impossible to read in the pdf file.*

R3R#2: Please note that the figures in the PDF for review are of a lower quality than those in the published paper. For our figures (Fig 1 and Fig 2), we followed the submission guidelines for authors. Therefore, the quality of the submitted figures would not be an issue. However, we present Figure 1 below for review.

Figure 1. (A) Transmittance of gray photochromic lenses in the colored (T_0_) and colorless state (T_∞_) at the warm temperature.

Figure 1. (B) Transmittance of brown photochromic lenses at the colored (T_0_) and colorless state (T_∞_) at the warm temperature.

Figure 1. (C) Transmittance of gray photochromic lenses at the colored (T_0_) and colorless state (T_∞_) at the cold temperature.

Figure 1. (D) Transmittance of brown photochromic lenses at the colored (T_0_) and colorless state (T_∞_) at the cold temperature.

*Reviewer #2: The fading kinetics of the photochromic lenses was assumed to follow a mono-exponential behavior which is a big simplification since two colored species are formed. From fig 2A one can see the effects of this simplification: the data does not follow a straight line. Fig 2b,c,d show smaller deviations because the authors collected only 4 points, which is rather low. This aspect should be commented.*

R4R#2: “The figures show a linearity between time, and the logarithm of absorbance showed the following order: cold temperature at λ_max2_, warm temperature at λ_max2_, cold temperature at λ_max1_, and warm temperature at λ_max1_. Although the evaluation of linearity is limited by the measurement of only a few points, a lower linearity in the photochromic lenses, including NKT gray, existed at the warm temperature at λ_max1_. This linearity is, in part, due to the scanning range (from 780 to 380 nm) over a long period (0–360 sec). Scanning may cause a difference between the time-intervals at each λ_max1_, and the measurement over a long relative to a short time may be also influence the linearity.” was added on page 14, lines 302–310. The limitations of this study for determining the fading rate based on the criteria of λ_max1_, λ_max2_, and the mean of 380–780 nm are presented in Table 6.

*Reviewer #2: The experiments were conducted at 21 degrees to simulate the Korean summer and at 6 degrees to simulate the Korean winter (-6-7 degree). Although I understand the difficulties in performing this experiments at -6 degrees, the authors should comment on the expected effect from going from 6 to -6 degrees.*

R5R#2: “Although our experiment was not performed below 0 °C, the fading rate at −6 °C can be approximately 2–3 times longer than that at 6 ± 2 °C when considering the warm versus cold temperature ratios for k_1_ and k_2_ in Table 4, as also shown in the study of Chu [36].” was added on page 21, lines 457–461.

*Reviewer #2: Some of the lenses show a big difference in the lifetime measured at lmax1 or lmax 2. This may lead to a color change during decoloration. For instance, DMP brown has a lifetime of 198 s at 456 nm and 51 s at 584 nm. This is quite different and must be commented. The same for RDP brown.*

R6R#2: “Large differences in the half-life time measured at λ_max1_ and λ_max2_ were evident for RDP brown, DMP brown, HYS gray, and HYS brown. The t_2(1/2)_ at the warm temperature was shorter than t_1(1/2)_ in RDP brown and DMP brown, having a higher transmittance (low absorbance) (Fig. 1B). These differences may be due to the differences between λ_max1_ and λ_max2_ in the scanning range (from 780 to 380 nm). However, t_1(1/2)_ at the cold temperature was shorter than t_2(1/2)_ in HYS gray and HYS brown. These lenses also showed a low transmittance (high absorbance) at the cold temperature (Fig. 1A and 2B). The differences were more noticeable at the cold than at the warm temperature, which may be due to the properties of the photochromic materials [26, 35] and the matrix polarity [30, 37] in the lenses. However, in the present study, this is difficult to explain because there was no information on the composition of HYS gray and brown, such as related to photochromic dyes and the matrix.” was added on page 21, lines 461–473.

*Reviewer #2: The conclusions of this work are rather vague. That the fading kinetics are temperature depend everyone knows. For instance, the authors don’t identify which lenses show the lowest temperature dependency, which one maintains its color shade, or not. After a so long statistical study, the conclusions are a deception. Since the goal was to analyze the marketed lenses the study should be more objective especially the conclusions.*

R7R#2: “The temperature dependence was lower for RDP gray, RDP brown, and DMP brown for both k_1_ and k_2_, and was higher for HYS gray and HYS brown for both k_1_ and k_2_. It was high for NKT brown for k_1_ and DMT gray for k_2_. The relationship between the ratio of the warm to the cold temperature for k_1_ and k_2_ was significant for Spearman’s rank correlation (ρ = 0.705, p = 0.019). ” was added on page 22, lines 473–477.

*Reviewer #2: The conclusion could include a table or a paragraph to answer all the topics written in the last paragraph of the abstract:*

*Abstract: “There were significant differences in the optical properties of photochromic lenses in terms of showing absorbance at a shorter wavelength, lower transmittance, higher optical density, higher optical blocking % ratio, and higher luminous transmittance in cold temperature than in warm temperature. Hence, it is necessary to provide consumers with information on photochromic optical properties including …”*

R8R#2: The “In summary” paragraph in the Discussion section was compared with the “Conclusion” in the Abstract as below (Figure R3). As a result, the highlights of this study were included in both the Abstract and Conclusions sections.

Figure R3. Comparison of between the ‘In summary’ paragraph in the Discussion and the ‘Conclusion’ phrase in the Abstract

========================

*Reviewer #3: The authors present a study on the kinetics of commercially available photochromic spectacle lenses. The study seems to be well conducted and the results have been clearly presented. The underlying data has been made available.
Specific comments:

1. The statement in the abstract does not seem to be correct "... properties at cold than at warm temperatures were 11.5% lower for transmittance at the colored state, 1.4 times higher for optical density". According to the data this should be "1.4 times higher for the change in optical density (from faded to colored state).
The corresponding text in the manuscript main text should be revised accordingly*

R1R#3: We revised the text according to the reviewer’s comment.

*Reviewer #3: 2. page 5, line 107: "All were middle index" does not sound very scientific. As according to the given refractive indices of the lenses, most of the lenses are most likely made of CR-39, the standard material. I'd suggest the following: "All lenses had a refractive index of 1.5-1.54 (standard material)".*

R2R#3: We revised the text on page 5, line 108 to “All lenses had a refractive index of 1.5–1.55 (middle index)” according to the reviewer’s comment.

*Reviewer #3: 3. page 6, line 128: Please add some more data on the spectrometer, what kind of light source was used?*

R3R#3: The phrase “with 190 to 900 nm wavelength, a deuterium and tungsten lamp as the light source, and a spectral bandwidth of 0.1–5.0 nm.” was added on page 6, line 130.

*Reviewer #3: 4. page 9, line 193: is this correct that there is three times t1(1/2) ?? Does this make any sense?*
R4R#3: The phrase “t_1(1/2)_, t_1(1/2)_, and t_1(1/2)_” has been revised to “t_1(1/2)_, t_2(1/2)_, and t_3(1/2)_” on page 9, line 198.

*Reviewer #3: 5. The fact that photochromism shows different behaviour related to the ambient temperature has been shown previously. The authors may want to include the following literature to provide a better unterstanding on the thermal effects in photochromic materials:
R. J. Araujo, "Kinetics of Bleaching of Photochromic Glass," Appl. Opt. 7, 781-786 (1968)
D. C. Look and W. L. Johnson, "Transmittance of photochromic glass at environmental extremes," Appl. Opt. 18, 595-597 (1979)*

R5R#3: We revised the manuscript according to the reviewers’ comments.

We inserted on page 5, line 91, reference [23]: 23. Look DC, Johnson WL. Transmittance of photochromic glass at environmental extremes. Appl Opt. 2006; 18(5):595-597. doi: 10.1364/AO.18.000595

We inserted on page 8, line 172, reference [27]: 27. Araujo RJ. Kinetics of bleaching of photochromic glass. Appl Opt. 1968; 7(5):781-786. doi: 10.1364/AO.7.000781

*Reviewer #3: 6. The authors should discuss, that the measurements are affected by the fact that the activation has been performed outside the spectrometer and the lens was already deactivating/fading during spectrometer measurement. This may affect the results considering that the scanning speed of the spectrometer (500 nm/min) is relatively long. Given that only a VIS-spectrum was measured (bandwidth ~ 380 nm between 380 nm and 760 nm), the measurement would take about 46 seconds which is nearly half of t(1/2) at least for the measurement at the warm state*

R6R#3: We fully agree with the reviewer’s comments. To reduce systemic errors, including an error due to time delay, we performed the activation after the simulation so that the activated photochromic lenses were placed in the spectrometer as quickly as possible. We pointed out, in lines 496–498 on page 23, the limitations of the study as follows. “the delay time in transferring the activated lens to the spectrophotometer was not considered, but a relative comparison of the characteristics of each fading rate is considered possible.” The characteristics for the fading rate (t_1(1/2),_ t_2(1/2)_) determined in the scanning process are discussed in Table 6.

*Reviewer #3: 7. In general it would be extremel interesting to also have the change in the UV transmittance/absorbance of the lenses (< 380 nm). Are these data available?*

R7R#3: The UV region (<380 nm) outside the visible region was not considered due to a blocking under 360 nm in middle index lenses (CR-39 or ADC = allyldiglycol carbonate).

Reference:

34. Ross Iii DF. Ophthalmic lenses: accurately characterizing transmittance of photochromic and other common lens materials. Appl Opt. 1991; 30(25): 3673–3577.

*Good luck with your manuscript!*

*6. PLOS authors have the option to publish the peer review history of their article (*[*what does this mean?*](https://journals.plos.org/plosone/s/editorial-and-peer-review-process#loc-peer-review-history)*). If published, this will include your full peer review and any attached files.

If you choose “no”, your identity will remain anonymous but your review may still be made public.*

***Do you want your identity to be public for this peer review?*** *For information about this choice, including consent withdrawal, please see our* [*Privacy Policy*](https://www.plos.org/privacy-policy)*.

Reviewer #1: No
Reviewer #2: No
Reviewer #3: No*

Authors’ Response: N/A

- The end of response to reviewers –
